# Quantitative Detection of Biological Nanovesicles in Drops of Saliva Using Microcantilevers

**DOI:** 10.1021/acsami.3c12035

**Published:** 2023-12-29

**Authors:** Clodomiro Cafolla, James Philpott-Robson, Aaron Elbourne, Kislon Voïtchovsky

**Affiliations:** † Department of Physics, Durham University, Durham DH1 3LE, U.K.; ‡ School of Science, STEM College, RMIT University, Melbourne, VIC 3001, Australia

**Keywords:** microcantilever, non-Newtoninan fluid, saliva, detection, microrheology, biofouling, cancer, extracellular nanovesicles

## Abstract

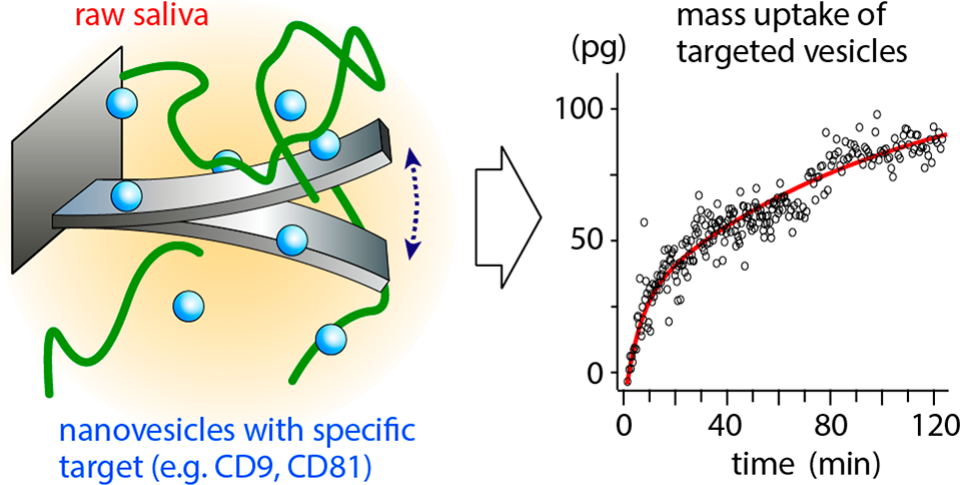

Extracellular nanovesicles (EVs) are lipid-based vesicles secreted by cells and are present in all bodily fluids. They play a central role in communication between distant cells and have been proposed as potential indicators for the early detection of a wide range of diseases, including different types of cancer. However, reliable quantification of a specific subpopulation of EVs remains challenging. The process is typically lengthy and costly and requires purification of relatively large quantities of biopsy samples. Here, we show that microcantilevers operated with sufficiently small vibration amplitudes can successfully quantify a specific subpopulation of EVs directly from a drop (0.1 mL) of unprocessed saliva in less than 20 min. Being a complex fluid, saliva is highly non-Newtonian, normally precluding mechanical sensing. With a combination of standard rheology and microrheology, we demonstrate that the non-Newtonian properties are scale-dependent, enabling microcantilever measurements with a sensitivity identical to that in pure water when operating at the nanoscale. We also address the problem of unwanted sensor biofouling by using a zwitterionic coating, allowing efficient quantification of EVs at concentrations down to 0.1 μg/mL, based on immunorecognition of the EVs’ surface proteins. We benchmark the technique on model EVs and illustrate its potential by quantifying populations of natural EVs commonly present in human saliva. The method effectively bypasses the difficulty of targeted detection in non-Newtonian fluids and could be used for various applications, from the detection of EVs and viruses in bodily fluids to the detection of molecular clusters or nanoparticles in other complex fluids.

## Introduction

The ability to achieve quantitative detection of specific molecules, chemical markers, or nanoscale assemblies in bodily fluids is at the heart of medical laboratory diagnostics.^1^ Examples range from early detection of toxins,^2^ microbiological agents,^3^ or tumoral biomarkers,^4^ to the routine monitoring of patients with chronic diseases such as diabetes,^5^ thrombophilia,^6^ or myelodysplastic syndromes.^7^ Typical diagnostic bioassays comprise enzyme immunohistochemistry,^8^ liquid chromatography,^6^ protein and genetic electrophoresis,^9^ and blood cultures.^10^ While these methods offer good levels of sensitivity and specificity, they suffer from several drawbacks. First, they tend to be time-consuming and require purification and preparation steps, which can potentially alter the antigen under investigation and affect the detection itself.^9^ Second, they are often expensive due to their complexity and the need for reagents or advanced experimental setup. Finally, they tend to require relatively large volumes of bodily fluids. Part of the problem comes from the complexity of bodily fluids, which contain a wide size and compositional range of molecules, proteins, biopolymers, and cells, often at concentrations significantly larger than that of the desired detection target. Additionally, most bodily fluids are highly non-Newtonian,^11^ and their inherent compositional heterogeneity renders physical detection methods such as mechanical resonators^12^ or nanofluidics-based approaches^13^ challenging. As a result, detection methods operating directly into raw bodily fluids are sparse, especially when aiming to detect and quantify a specific target within that fluid. Being able to operate with small quantities of unprocessed bodily fluids could prove a game changer for detection and medical prognosis, potentially cutting costs and diagnosis time as well as offering measurements better reflecting the natural environment of the desired target.

Here, we show that quantitative measurements can be achieved in single drops of saliva by combining immunorecognition with mechanical detection optimized to operate on the correct scale. Saliva is arguably one of the most challenging bodily fluids to operate in given the presence of large biopolymers forming gel–liquid structures, but this can be overcome by accordingly adapting the mechanical sensing. The choice of saliva is also motivated by its potential for noninvasive and real-time diagnostics of infective and neoplastic diseases.^14,15^ To illustrate the capabilities of our method, we target the model and native extracellular nanovesicles (EVs). EVs are small (30–300 nm) phospholipid-based vesicles present in most bodily fluids including blood, saliva, and urine.^16,17^ They are secreted by cells into the surrounding connective matrix and are naturally used as vehicles to cargo small molecules, proteins, and nucleic acids between distant cells and throughout the body.^16^ EVs play a key role as autocrine and paracrine signals^16^ regulating multiple cellular functions from growth and apoptosis^18^ to gene expression and antigen presentation.^19^ They have been suggested as biomarkers for early detection and monitoring of various diseases including cancer,^20,21^ diabetes and metabolic conditions,^5,22^ neurodegenerative pathology, and viral or microbiological infections.^23^ Several pathologies promote the release of specific EVs with a unique combination of antigens in terms of both type and concentration.^17^ However, routine use of EVs in diagnostics is currently still limited by the costs and slowness previously highlighted. Additionally, existing characterization methods tend to focus on genetics and proteomics and require relatively expensive purification and concentration of milliliters of bodily fluids^24,25^ with no accepted standards.

Using vibrating microcantilevers suitably functionalized, we are able to bypass these issues and quantify specific EV populations directly into saliva. Vibrating microcantilevers have long been used as biosensors,^26,27^ including proposed approaches for cancer detection,^12^ but operating in liquids tends to limit the sensitivity of the technique,^28^ and most applications rely on sensing in vacuum, air, or purified solutions.^26^ More recent developments in the field of nanomechanical systems have focused on optomechanical resonators^29^ or sophisticated bespoke systems^30^ to achieve high levels of sensitivity. However, operating directly in complex biological fluids remains a significant challenge, limiting applicability, use, and direct diagnostics. Here, we show that saliva exhibits scale-dependent viscoelasticity, a property that we exploit to operate in raw saliva with a sensitivity comparable to that achieved in pure water. The proposed approach can be upscaled, parallelized, and in principle applied for the detection of a wide range of targets directly in complex fluids.

## Experimental Section

Ultrapure water was purchased from Water AnalaR NORMAPUR, VWR International Ltd., Leicestershire, UK. For all of the experiments using saliva, fresh samples were obtained from healthy volunteers (two of the authors) and used on the same day. The saliva samples were collected in the morning between 7:00 am–9:00 am after overnight fasting, directly into a sterile glass vial, and used without any further processing or purification. The samples not immediately used were kept at 5 °C (measurements conducted later in the day) and warmed to the measuring temperature (25 °C) immediately before use, taking care to keep the sample homogeneous. For the experiments using model EVs, the desired quantity of EVs was added to the sample, which was then homogenized in a mild sonication bath (see details in the Model EVs section hereafter).

### Shear Rheometry

Shear rheometry was performed using a commercial Advanced Rheometer model AR 2000 (TA Instrument, New Castle, DE, USA), equipped with 8 mm parallel plates and an environmental test chamber under nitrogen gas. The fluids were compressed between the parallel plates under atmospheric pressure until a gap of approximately 1 mm thickness and a small normal force was registered by the rheometer. To determine the full rheological response, oscillatory tests were performed at angular frequencies between 0.1 and 600 rad/s, and with strain amplitudes of 1%, after examination of the dynamic strain sweep as a function of frequency. The temperature was kept constant at 25 °C.

### Microrheology

Microrheology measurements were performed using a Malvern Zetasizer NanoZSP (Malvern Panalytical, Worcestershire, UK). The tracer particles were silica nanospheres with nominal diameters of 50, 100, and 300 nm monodispersed in water with a concentration of 10 mg/mL (nanoComposix, San Diego, CA, USA). The silica particles were then diluted in water or saliva to a final concentration of 0.1 mg/mL and tip-sonicated for 45 s to remove any aggregates. Before microrheological measurements were conducted, standard dynamic light scattering (DLS) ensured a monodisperse distribution of the particles.

### Lipid-Coating of the Tracers

Lipid-coating of the tracers was performed by incubating small unilamellar lipid vesicles (SUVs) of 1,2-dioleoyl-*sn*-glycero-3-phosphocholine (DOPC) into a PBS solution (137 mM NaCl, 2.7 mM KCl, 10 mM Na_2_HPO_4_, and 1.8 mM KH_2_PO_4_ at pH 7.4) containing the dissolved silica tracers. To ensure full coating, we estimated the total surface area of the tracers in solution and used a 10-fold excess of fluid lipid vesicles (in terms of total bilayer area) adsorbing onto the silica particles. The particles were then diluted in PBS to the desired concentrations for the experiment. DOPC was purchased in liquid form, dissolved in chloroform (Avanti Polar Lipids, AL, USA), and used without any further purification. After chloroform evaporation in a vacuum overnight, lipids were resuspended in a PBS solution at a final concentration of 10 mg/mL. PBS solution was produced using preprepared tablets (Sigma-Aldrich, St Louis, MO, USA). SUVs of diameter ∼100 nm were obtained by bath-sonicating the lipid solution at 25 °C for 10 min to produce a uniformly clear solution, followed by extrusion through a 100 nm filter (WhatMan, Sigma-Aldrich) with at least 31 passes, and then used immediately.

### Model EVs

Model EVs were prepared with a biotinylated lipid mixture. The lipid mixture comprised 99.5% dipalmitoylphosphatidylcholine (DPPC) and 0.5% biotinylated-DPPE (1,2-dipalmitoyl-*sn*-glycero-3-phosphoethanolamine). Both lipids were purchased from Avanti Polar Lipids, AL, USA, and mixed to the desired ratio in chloroform. The chloroform was then evaporated in a vacuum overnight. After resuspending the lipids in PBS and bath-sonicating them at 60 °C for 15 min, SUVs were then prepared by extrusion through a 100 nm filter. The desired proportion of model EVs was then added to the saliva samples and bath-sonicated for 5 min before use.

### Cantilever Functionalization

Cantilever functionalization was performed using the same lipid mixture used for the model EVs (99.5% DPPC + 0.5% biotinylated DPPE). Before functionalization, the cantilevers underwent thorough cleaning procedures to ensure the removal of any potential contaminants.^31−33^ The cantilevers were immersed in a bath of ultrapure water, followed by propan-2-ol (Merck Millipore, Billerica, MA, USA), and finally ultrapure water, for 60 min at each step. The propan-2-ol was used as purchased without further purification.

The cantilevers were then exposed to low-pressure air plasma, at a pressure of 1 mbar and power of 300 W (VacuLAB Plasma Treater, Tantec) for 30 s. Plasma-oxidation increased the hydrophilicity of the cantilevers and removed unwanted carbon contaminants. A drop of SUVs (150 μL) with a concentration of 1 mg/mL was drop-cast on the cantilever. After a 20 min incubation, the cantilevers were gently rinsed with freshly prepared PBS and left soaking in clean PBS for 1 h to ensure removal of any excess nonadsorbed vesicles. After this, the cantilevers were rinsed again with PBS.

The cantilevers were then further functionalized using streptavidin (Thermofisher, UK), which acted as a bridge between the two biotinylated lipid bilayers forming the EVs’ surface and the cantilever coating. The streptavidin functionalization was performed by first soaking the probes in a solution containing 0.1 mg/mL streptavidin in PBS. After incubation for 1.30 h, the cantilevers were gently rinsed with PBS and then left soaking in clean PBS for 1 h.

The cantilevers used in the detection of naturally occurring EVs needed a further functionalization step. This involved the binding of anti-CD9 and anti-CD81 monoclonal antibodies to the streptavidin functionalized cantilevers. The antibodies were obtained by recombinant DNA technique, with a human species reactivity and purchased from ABCAM, UK. The binding of the antibodies to the streptavidin was performed by biotinylation conjugation using the specific Biotin Conjugation Kit (Fast, Type A) Lightning-Link (ABCAM, UK). The cantilevers used for the negative controls were functionalized with streptavidin (Results section: Testing the Method with Model EVs) and pure DPPC (Results section: Detecting Specific Natural EV Subpopulations in Human Saliva). The design of suitable controls is further discussed in the Supporting Information.

### Atomic Force Microscopy (AFM)

**Figure 1:**
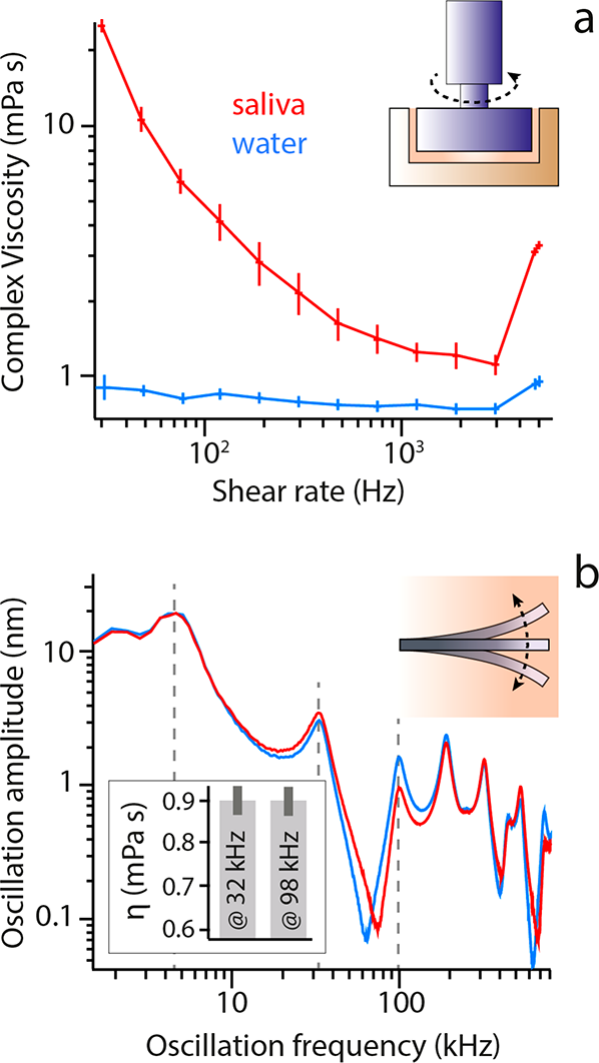
Probing saliva’s viscosity at different length scales. (**a**) Macroscopic standard shear rheological measurements highlight the non-Newtonian behavior of raw saliva (red) in comparison with pure water (blue). (**b**) The nanoscale viscoelastic behavior of raw saliva is also probed using a vibrating microcantilever operated with an AFM. The viscosity of the liquid surrounding the cantilever can be quantified from the frequency shift of the vibration resonances,^41^ with the method applied here to comparatively probe the viscosity of water and of raw saliva. No shift is observed between water and saliva (dashed lines), and the derived viscosities for saliva are identical to water within error (inset in **b**) (see Experimental Section and Figure S1 for more details). The microcantilever measurements were performed using a commercial microcantilever (Olympus, OMCL-RC800 PSA) at 25.0 ± 0.1 °C, with the probe fully immersed in liquid.

The experiments on the dynamic response of vibrating microcantilevers in water and saliva were conducted using a commercial Cypher ES AFM (Oxford Instruments, CA, USA) instrument equipped with temperature control. We used two types of commercial cantilevers with each cantilever calibrated using its thermal spectrum.^34^ Initial experiments (Figure 1) were conducted with OMCL-RC800PSA silicon oxide cantilevers (Olympus, Japan) that exhibit a stiffness of 0.1–0.2 N m^–1^ and a resonance frequency of 20 ± 5 kHz in air. All the quantitative measurements were conducted with stiffer and shorter AC55-TS silicon oxide cantilevers (Olympus, Japan), which exhibit a typical flexural stiffness of 50 ± 5 nN/nm and a resonance frequency of 1600 ± 300 kHz in air. High-quality V1 muscovite mica discs (SPI supplies, West Chester, PA, USA) acted as a substrate where to deposit the fluid of interest; 150 μL of the fluid of interest was deposited on the mica substrate. All the experiments were performed at 25.0 °C to ensure thermal stability.^35,36^ Thermal equilibrium is achieved ensuring that the cooling/heating rate of the temperature control system within the AFM is constant for at least 20 min. After thermal equilibrium was achieved, the cantilever was then fully immersed in the fluid, with its motion driven by photothermal excitation.

## Results

### Scale-Dependence of Saliva’s Viscoelasticity

Saliva, like most bodily fluids, is a complex fluid and exhibits a nonlinear viscoelastic behavior upon applied mechanical strain.^37^ Although the specific properties of saliva are person-, time-, and condition-dependent,^38,39^ typical rheological measurements reveal viscosities 1 order of magnitude larger than for water in identical conditions (Figure 1). This is due to the presence of numerous large biopolymers which underpin saliva’s non-Newtonian behavior,^39^ even preventing flow through a 200 nm filter.^40^ At the macroscopic scale, the viscoelastic differences between pure water and saliva are obvious at all accessible shear rates (Figure 1a) and consistent with previous studies.^39^ At the nanoscale, viscomechanical sensing can be performed with vibrating microresonators.^29^ Considering the rheological findings, mechanical sensing in saliva could be expected to induce a significant reduction in the frequency and amplitude of any vibrating resonators compared to pure water. Interestingly, this is not necessarily the case, as illustrated here using a vibrating commercial microcantilever immersed into either water or saliva and operated with an AFM (Figure 1b). We could not observe any significant variation between the measurements obtained in pure water and saliva for oscillation amplitudes smaller than ∼20 nm: the amplitude and frequency of the microcantilever’s resonances remain broadly unchanged (Figures 1b and S1 for more details). This suggests that the cantilever experiences a nearly identical environment between water and saliva. To further quantify this observation, we use the resonance frequencies of the vibrating microcantilever to determine the viscosity of saliva as experienced at the probed vibration frequencies.^41^ A constant viscosity value identical to that of pure water within error was found at both 32 and 98 kHz (second and third resonances, inset Figure 1b, see also Supporting Information section 1 for further details).

A careful comparison of all the resonances in water and in saliva (Figures 1b and S1) indicates that the same conclusion holds, regardless of the frequency probed: no systematic frequency shifts to lower values are observed from water to saliva as would have been expected for a higher viscosity liquid.^41^ To rationalize this apparently counterintuitive finding, it is necessary to consider the structure of saliva across scales. Saliva can be understood as a (bio)polymeric mesh, filled primarily with water.^42^ Proteins and small biological objects such as EVs are dissolved into the water and fill the mesh structure where they can diffuse freely within gaps. The mesh itself is not a static cross-linked structure, but any structural rearrangement of the polymeric network is significantly slower than that of the water it contains, hence conferring saliva its non-Newtonian macroscopic behavior. Because standard rheological measurements are macroscopic, they are dominated by the viscoelastic behavior of the polymeric mesh under strain, with its pronounced viscous and elastic responses (see Supporting Information Figure S2). In contrast, microcantilevers operate with nanoscale oscillation amplitudes comparable in size to the gaps naturally existing in the polymeric mesh. If the oscillation amplitude *A* of the vibrating cantilever is smaller than the average gap size *S* of the mesh, the cantilever primarily experiences the viscous water diffusing within the mesh, with limited impact of the polymers on the measurements.

It is important to keep in mind that, while useful, the idea of saliva as a mesh is a simplification of reality, and no single value of *S* exists. Instead, *S* can be understood as an effective size marking the transition from a polymer-dominated (at larger scale) to a water-dominated viscoelastic behavior at smaller scale. This scale-dependent viscoelasticity has previously been reported^43^ in complex fluids and is likely common given their hierarchical structure. Here, we exploit this scale-dependence to enhance the detection capability of microcantilevers operating directly into saliva: if the oscillation amplitudes are small enough, the sensing microcantilever effectively operates in a simple aqueous solution, where a significantly better signal-to-noise ratio can be achieved.

**Figure 2:**
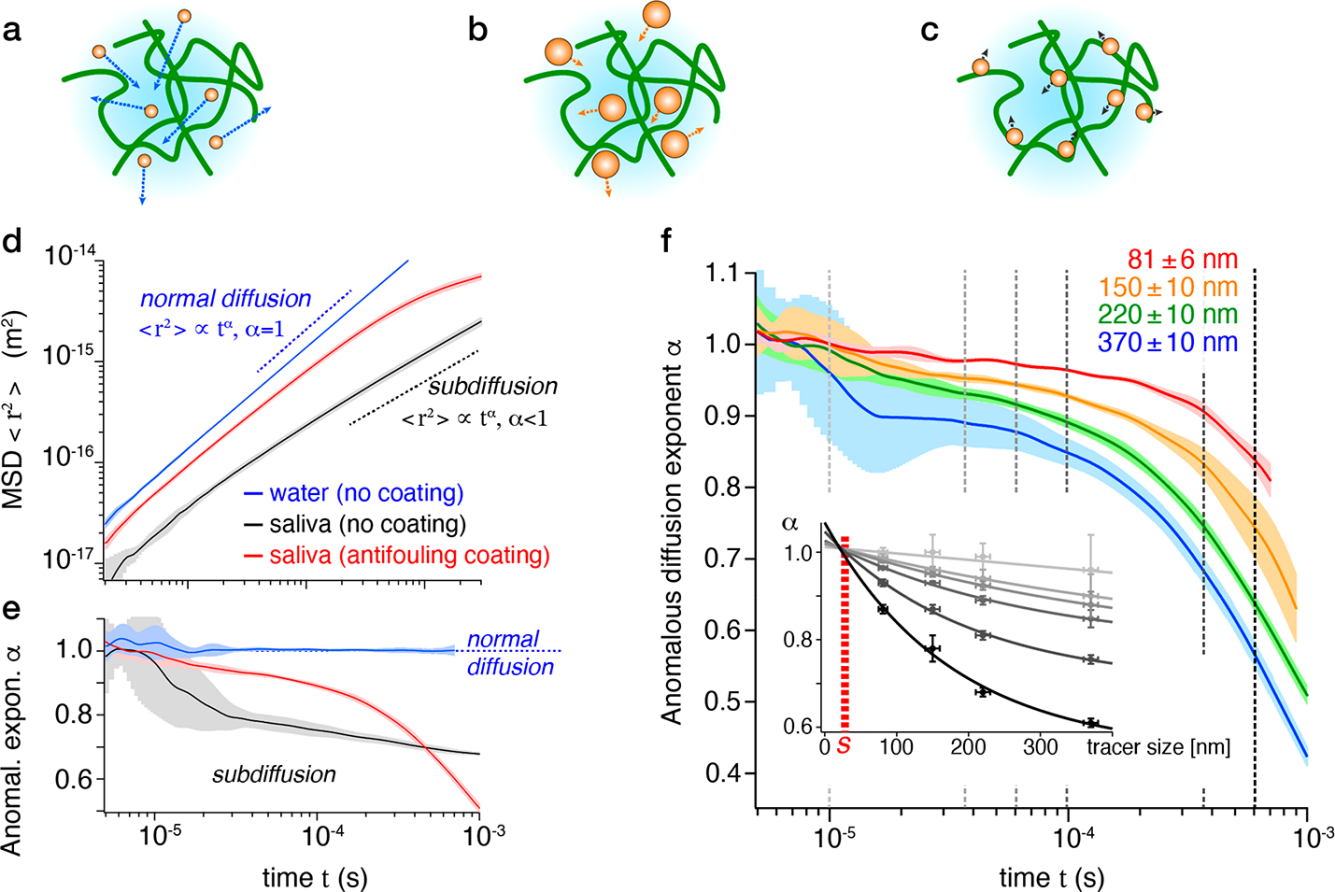
Passive microrheology of raw saliva with silica tracers. A cartoon representation of the system (**a**–**c**) illustrates the fact that smaller tracers (**a**) can diffuse more easily through the mesh formed by saliva compared to larger tracers (**b**). However, this assumes a limited interaction of the tracer particles with the mesh. Otherwise, interactions with the mesh reduce mobility (**c**) and affect smaller particles relatively more due to their larger surface to volume ratio. (**d**) Example of a measurements with a 73 ± 6 nm tracer (without coating) showing the MSD <*r*^2^> as a function of time *t* on a log–log plot. In pure water, <*r*^2^> ∝ *t* indicating normal Brownian diffusion. In saliva, <*r*^2^> ∝ *t*^*α*^ with α < 1, the so-called anomalous diffusion exponent indicating subdiffusion. (**e**) The evolution of α at different time scales highlighting differences for water (α ≈ 1, blue curve) and saliva (α < 1) with and without a zwitterionic antifouling coating on the tracer (red and black curves, respectively). Over a short observational time scale (<10 μs), the tracers are able to freely diffuse unhindered. Over longer time scales or for larger tracers, the impact of interactions with saliva components tends to hamper the diffusion. Measurements >0.5 ms are less reliable, being close to the tracking limit of the equipment. Here, this is visible in α becoming lower for coated than uncoated tracers, despite a significantly larger MSD. (**f**) Time evolution of α for tracers of different sizes. Comparison of α vs tracer size at selected times (10 μs, 25 μs, 50 μs, 0.1 ms, 0.25 ms, and 0.5 ms, vertical dashed lines) suggests consistent unhindered diffusion for tracers <25 nm (inset). All the particles’ diameters are measured by DLS using the same setup as for the microrheology (see Figure S3 and Table S1 within section 3 of the Supporting Information).

To achieve enhanced microcantilever detection in saliva, we first set out to objectively identify the value of *S*. The fact that saliva cannot flow through a ∼200 nm filter while water can easily pass through it^13,40^ suggests that *S* < 200 nm. This is consistent with the fact that no significant differences between water and saliva could be observed for immersed microcantilevers vibrating with amplitudes below ∼20 nm (Figure 1b), suggesting *S* to be in the 20–200 nm range. To independently quantify *S*, we used microrheology^44^ with tracers ranging from 70 to 370 nm (see Supporting Information section 3 and Table S1). If the tracers are able to diffuse freely within the mesh (Figure 2a), their mean square displacement (MSD) is expected to follow standard Brownian diffusion and grow linearly over time.^44,45^ In contrast, if the tracers’ diffusion is hampered by the mesh (Figure 2b,c), a subdiffusive^45^ behavior is expected whereby the MSD is proportional to the time at a power α < 1. It is therefore convenient to track the anomalous diffusion exponent α for each tracer in order to distinguish “free” (α = 1) from mesh-hindered (α < 1) diffusion. An example of microrheological measurements is shown in Figure 2d,e, carried out with silica spherical nanoparticles as tracers. The average diameter of the particles is 73 ± 6 nm (50 nm nominal, see Table S1), and the use of silica tracers is motivated by the fact that the surface of the microcantilevers is primarily silica.

As immediately obvious from the measurements, when operating in saliva, it is crucial for the tracers to be coated with an antifouling layer so as to prevent nonspecific binding to saliva’s constituents. In principle, nonspecific binding can occur with salivary proteins (e.g., mucin fibers, lactoferrin, IgA), ions (calcium, phosphate, carbonate, and thiocyanate ions),^1^ and the biopolymeric mesh itself, resulting in a lower mobility of the tracers (Figure 2c). The issue of biofouling is common to most measurements in biologically active environments,^13^ often deteriorating the accuracy and precision of the measurements over time.^46^ Here, it must be addressed since the microcantilever-based detection strategy implicitly assumes that saliva’s biopolymers do not bind to the cantilever but rather move around it if occasionally disturbed. If the polymers attach to the cantilever, the latter becomes part of the mesh and hence primarily measures the mesh’s viscoelastic behavior, something we aim to prevent. To tackle this issue, we coat the tracers with a self-assembled zwitterionic lipid bilayer. The zwitterionic nature of the lipid headgroups significantly reduces unwanted interactions and enhances the tracers’ diffusion, as evidenced by the clear increase in α (black to red, Figure 2d,e). Coating with a zwitterionic bilayer is a simple and effective antifouling strategy and is systematically used hereafter. Practically, the coating also increases the measured diameter of the tracers by ∼8 nm, consistent with the size of two bilayers (Supplementary section 3 and Table S1).

Focusing on the evolution of the anomalous diffusion coefficient for different size tracers (Figure 2f), it is immediately clear that the larger the size of the tracer, the smaller the value of α. In other words, larger tracers are more hindered by the polymeric mesh, resulting in an accentuated subdiffusive behavior regardless of time. By plotting the α value as a function of the tracers’ size at set times, it is possible to infer the tracer size *S* which would satisfy α = 1 (Figure 2f inset). We find *S* = 25 ± 10 nm regardless of the time considered. This provides an objective estimate for *S*, indicating that smaller objects can, on average, diffuse freely through saliva as if it were a purely Newtonian fluid.

### Testing the Method with Model EVs

Based on the microrheology results, we use microcantilevers coated with an antifouling zwitterionic layer and oscillating with an amplitude smaller than *S* = 25 nm. In practice, the smaller the oscillation amplitude, the better, providing a sufficient signal-to-noise ratio. This is typically achieved using microcantilevers as small and stiff as possible, thereby ensuring a comparatively high resonance frequency and quality factor (see Supporting Information section 4) and hence sensitivity. Here, we use Olympus AC55 cantilevers (see Experimental Section) which offer some of the highest resonance frequencies and quality factors among commercially available levers.

**Figure 3:**
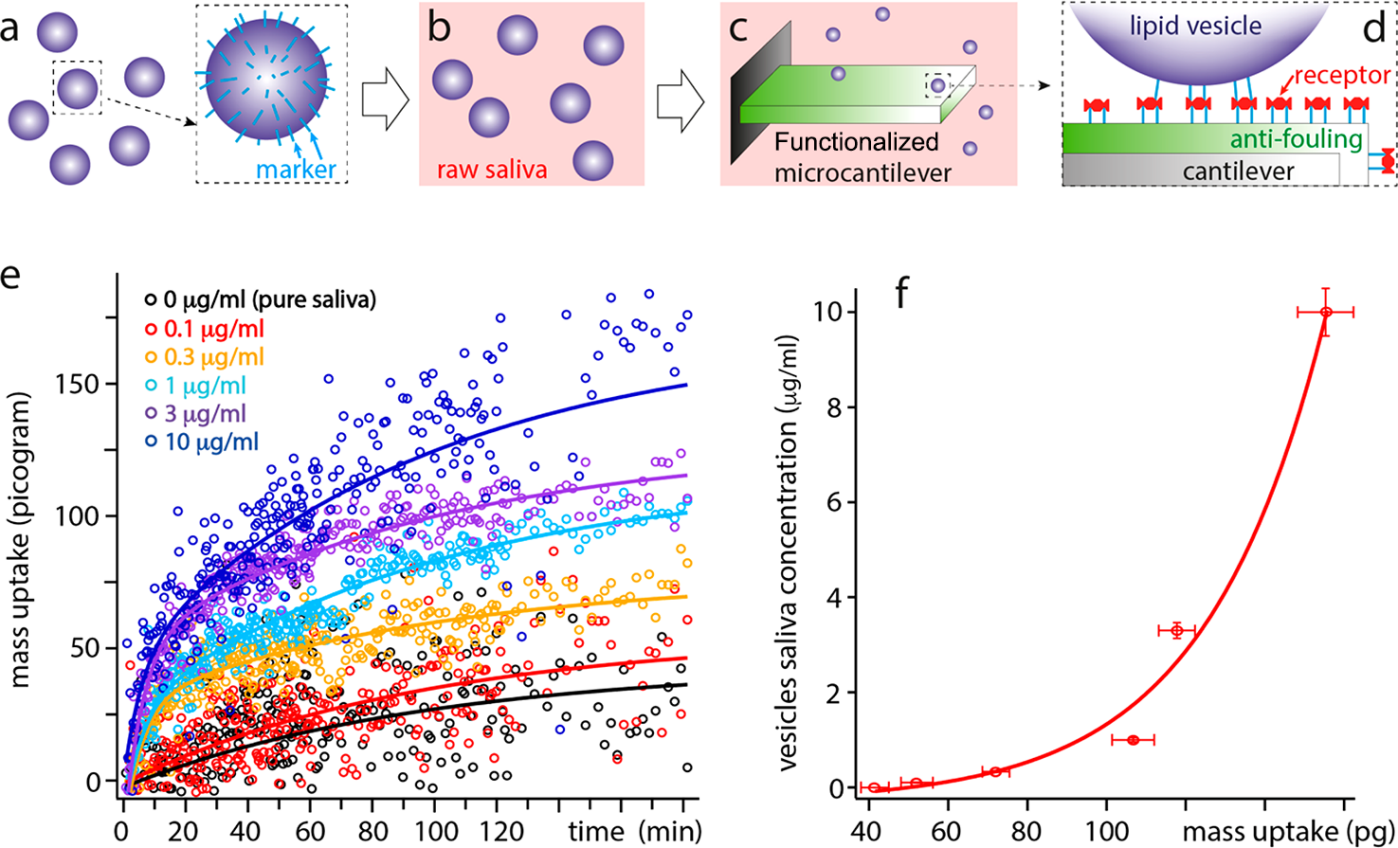
Characterization and testing of the proposed approach for targeted detection of specific EV subpopulations directly in raw saliva. The testing is carried out with model EVs composed of DPPC with 0.5% biotinylated lipids acting as surface markers (**a**) and dissolved into saliva (5% of the total volume from a phosphate buffer saline solution (**b**)). The cantilever is coated with a DPPC bilayer containing 0.5% biotinylated headgroups (**c**), preventing biofouling while ensuring specific binding of the model EVs after further streptavidin functionalization (**d**). From the changes in the cantilever’s vibration amplitude, phase, and frequency, the total mass of the target EVs binding to the cantilever can be precisely quantified despite the saliva background (**e**). Experimental data are fitted globally with a double exponential function, imposing the same two time scales (τ_1_ and τ_2_) for all the experiments. The most important differences occur within the first 5–10 min (τ_1_ = 346 ± 56 s), with only the slower evolution (τ_2_ = 5781 ± 778 s) present at 0.1 μg/mL and in pure saliva. (**f**) The maximum mass uptake (added mass at time = ∞) derived from the fitting also exhibits a double exponential increase against the EV concentration in saliva. All of the measurements are conducted at 25.0 ± 0.1 °C. The error in concentration (**f**) is assumed to be 10%, likely an overestimate.

To validate the proposed approach, we conducted a set of experiments aiming to quantify the amount of synthetic model EVs dissolved into raw saliva. The interest of using model EVs is twofold: first, since the saliva sample is prepared with a known concentration of model EVs, it allows for independent determination of the setup sensitivity. The concentration of a specific native EV’s subpopulations in saliva varies between individuals^47^ and experiments,^47,48^ making any independent measurements highly challenging. Here, the model EVs occur precisely as one of these subpopulations but with a unique protein marker and a specific concentration. Second, a comparison of the known EV concentrations with the measured quantities allows for calibration of the setup. To best mimic natural EVs in size and composition, we create 100 nm gel-phase phospholipid (DPPC) vesicles, with 0.5% of the lipids exposing a tether biotin acting as a specific EV marker (Figure 3a). The model EVs, dissolved in a standard phosphate buffer saline (PBS) solution, are then mixed with the raw saliva to achieve the desired final concentration, but always ensuring that the EV solution represents only 5% of the total saliva volume to minimally affect saliva’s properties (Figure 3b).

The microcantilevers are functionalized with the same gel-phase phospholipid bilayer containing 0.5% of biotinylated lipid headgroups. In this configuration, 99.5% of the zwitterionic headgroups act as a relatively robust antifouling layer with the membrane in gel phase; the biotinylated headgroups can specifically bind the model EVs after further functionalization of the microcantilever with streptavidin (Figure 3c,d). An AFM is used to track any changes in the cantilever resonance over time, allowing for quantification of the mass uptake associated with specific EV binding to the cantilever (see Supporting Information sections 4–5 and Figures S4–S5 for more details).

The results show a clear sensitivity to the model EVs binding to the cantilever (Figure 3e), with meaningful measurements achieved at concentrations down to 0.3 μg/mL in a single drop (100 μL) of saliva. The sensitivity threshold appears to be around 0.3 μg/mL, where the readout becomes close to the control. While the natural concentration of native EVs in saliva is not known, various studies estimate a range 1 or 2 orders of magnitude greater than the present sensitivity achieved.^49,50^ Interestingly, a rapid uptake is visible over the first 5–10 min, followed by a slower uptake also present in the control experiment (pure saliva). This suggests that a quantitative readout is possible in less than 30 min despite the small sample volume and the absence of any sample preparation or conditioning of the sample. This compares favorably to the standard EV characterization methods based on affinity columns,^51^ provided no quantification of the EVs’ encapsulated cargo is needed.

A consistent analysis of the results was achieved by globally fitting all the experimental results with a double exponential and imposing the same two time scales for all the experiments (Figure 3f). The evolution of the mass uptake with time, *m*(*t*), is fitted with the following equation:
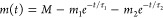
1where *M* is the maximum mass uptake in each experiment, and *m*_1_ and *m*_2_ are the concentration-dependent fitting coefficients associated with the global time scales τ_1_ and τ_2_. The use of a double exponential model to describe an adsorption process evolving over two distinct time scales is usually referred to as the Largitte double step kinetics model.^52^ The initial rapid uptake is not visible for the control and the lowest EV concentration (0.1 μg/mL), and the associated coefficient *m*_1_ are hence set to zero. When plotting *M* against the EV concentration *C* present in saliva at the start of the experiment (Figure 3f), a two-regime behavior emerges. Near the detection threshold, *M* increases rapidly with *C*, likely limited only by diffusion of the target EVs to the surface of the sensing cantilever. As more and more EVs get tethered to the surface, the binding rate decreases due to the fact that diffusing EVs need to find an uncovered region of the cantilever to bind. In this interpretation, this second regime dominates at larger *C* where it reduces the dependence of *M* over *C*, as visible in Figure 3e. Significantly, since the evolution of *M* with *C* is determined by the ability of EVs to bind to the microcantilever, Figure 3f effectively acts as a calibration curve for microcantilevers with the specific surface geometry used here. Additionally, since the technique requires only a drop of fluid, it can easily be multiplexed to simultaneously quantify multiple EV targets and improve accuracy.

### Detecting Specific Natural EV Subpopulations in Human Saliva

The results presented in Figure 3 validate the possibility of EV detection directly in bodily fluids using vibrating microcantilevers. However, in the absence of a precise reference or accepted standard for the EV populations naturally present inside bodily fluids, it is not yet obvious whether the method can achieve meaningful measurements of natural EV subpopulations. To test this, cantilevers identical to those used in Figure 3 are functionalized with antibodies able to selectively bind common natural markers. We selected members of the tetraspanin family (CD9 and CD81) as markers.^53,54^ CD9 and CD81 are cell surface glycoproteins which mediate a wide range of cell functions from B–T cell interactions to platelet activation^53^ and aggregation. Naturally occurring EVs with these markers are present in every bodily fluid of a healthy human being,^53^ but variations in their concentration could indicate neoplastic evolution^54^ or other diseases.^55^ Even if the range of concentration in healthy subjects remains to be determined with currently no accepted standard, the associated EVs have been suggested for early cancer diagnostics.^54,56^ Here, they are used as a generic test for the setup’s capabilities.

**Figure 4:**
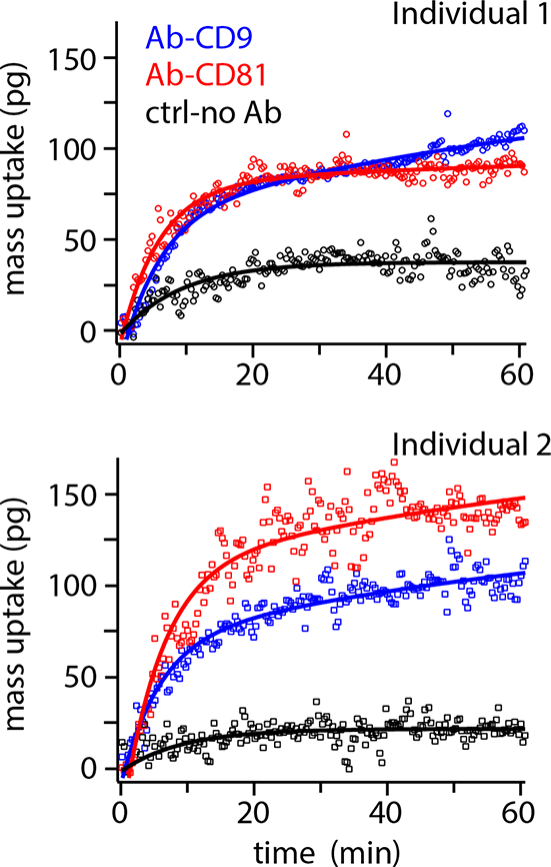
Detection of two natural EV subpopulations expressing CD9 and CD81 antigens on their surface, measured in saliva. The experiments are performed on a drop of unprocessed saliva samples from two healthy individuals. The cantilevers are functionalized as described in Figure 3 but with antibodies (Ab) to the targeted tetraspanin (see Experimental Section). The control probes (black) are coated only with the antifouling DPPC bilayer. As for model EVs, the mass uptake is rapid over the first 5–10 min and can be suitably analyzed imposing the same time scales as in Figure 3. The control is analyzed with a single exponential yielding in both cases a characteristic time scale of ∼550 s (see Supporting Information sections 6 for details). Experiments with anti-CD9 and anti-CD81 Abs were repeated twice for each sample with the average shown in the figure.

Figure 4 shows the results for the detection of natural EVs exhibiting CD9 or CD81 in the saliva of two different healthy individuals. The functionalization process is similar to that used for model EVs but with an additional step wherein a biotinylated version of the desired antibody is tethered to the exposed streptavidin receptor of the cantilever. Informed by the calibration experiment (Figure 3), the measurements are conducted over only 60 min and analyzed using the same double exponential fitting with the values of τ_1_ and τ_2_ imposed as the values found in Figure 3 (τ_1_ = 346 s, τ_2_ = 5781 s) except for the baseline fitted with a single exponential. The results show a clear difference in mass uptake between the baseline (black) and the cantilever functionalized with the tetraspanin antibodies (blue and red). The data are well fitted by the double exponential function with the imposed time scale, confirming the generality of the model in this configuration. Interestingly, the results highlight interindividual differences in the total content and ratio of EVs expressing CD9 and CD81 antigens. This further shows the potential of the proposed method to contribute to novel diagnostics and developing personalized medicine. Using the results from Figure 3f as a calibration, we estimate that the concentrations of the EVs expressing CD9 and CD81 antigens are respectively 10.3 ± 0.9 and 1.5 ± 0.2 μg/mL for individual 1 and 8.2 ± 1.1 and 36.1 ± 9.0 μg/mL for individual 2. We emphasize that these values cannot be independently verified in this study and were derived on the implicit assumptions that the setup behaves similarly as with model EVs, with a similar affinity for the microcantilever and without any uspecific fusion of the EVs with the cantilever’s antifouling lipid layer.^57^ These assumptions are not obvious and will require further work to confirm their reliability and benchmark the technique.^58^ Additionally, complications with the current multistep functionalization make it difficult to achieve consistent control measurements (see Supporting Information section 6 and Figures S6–7). Nevertheless, the present results show that the setup has the potential to detect and quantify specific subpopulations of EVs naturally occurring in human saliva.

## Discussion

Complex fluids are ubiquitous in nature from bodily fluids to water waste, oil reservoirs, lubricants, and food products. They play a key role in science and technology and are at the forefront of active research.^59^ Most complex fluids are composed of a mixture of macromolecules dissolved in a Newtonian fluid, with the dynamics of these macromolecules conferring the resulting solution its non-Newtonian behavior. Here, we show that saliva exhibits a scale-dependent viscoelastic behavior due to the finite size of the biopolymers forming a macromolecular mesh-like structure. At the nanometer level, saliva can be considered as a Newtonian fluid, whereas the non-Newtonian behavior emerges at a scale characteristic of the mesh. While we only investigate saliva in this study, the observed scale-dependent viscoelasticity is likely to be valid for many complex fluids that exhibit a similar structure over scales, but the relevant scales are likely to be fluid specific. For saliva alone, autoimmune pathologies altering the content of mucin fibers and antibodies in saliva and other bodily fluids^1^ could influence the diffusion length scale of tracers. This is also the case for any other complex fluids whose specific composition is expected to impact the diffusion length scale and, consequently, the mobility of sensing microcantilevers. Thus, the design of any detection methods for complex fluids should include a “calibration step” assessing this characteristic length scale.

We show that the scale-dependence can be exploited for conducting mechanical sensing directly in the complex fluid while retaining the signal-to-noise ratio normally only possible in simple Newtonian fluids. Calibration and testing of the setup with model lipid vesicles dissolved in raw saliva show a sensitivity in the picogram range, with the intrinsic detection noise level of the setup below this range. Based on the size and composition of the model EVs, we estimate their mass to be on the order of 0.001 pg (∼1 femtogram/model EV), suggesting an effective EV detection sensitivity in the range of 500–1000 EVs. The mass of natural EVs is likely significantly higher when taking into account the nucleic and proteic compounds, thereby increasing the sensitivity per EV. Additionally, the system used here solely relies on commercial equipment and could be significantly improved. First, the sensing cantilevers could be replaced by bespoke cantilevers with a geometry optimized for maximizing the sensing surface while retaining the ability to operate with small amplitudes and high frequencies. Second, the sensing could be developed with self-actuating microresonators, bypassing the need for the expensive laser system of the AFM. Finally, the process is suitable for parallelizing with multiple sensors operating over the same drop-size sample. This would open the possibility for simultaneous repeats and averaging as well as complementary detection of multiple targets, thereby improving the statistical accuracy and predictive power of the detection.

Detection of native EVs expressing the tetraspanin CD9 and CD81 antigens suggests that the setup is able to directly pick these subpopulations from saliva samples of healthy individuals. Here, tetraspanins are used as a test owing to their ubiquity in bodily fluids EVs. Several studies suggest that CD9 and CD81 expression may have a clinical significance in neoplastic diseases, but these are usually not seen as specific enough to represent a clear diagnostic tool.^60−65^ In fact, the results shown in Figure 4 indicate significant variations in the concentration detected between healthy individuals (see also Supporting Information section 7 and Figure S8 for more details). Variations may also occur over time for the same healthy individual, something we did not explore. Further work is needed to assess the suitability of the method on more specific targets, something potentially more challenging to achieve if the associated EV subpopulation is significantly smaller. Several antigens have already been identified for immunocapture of EVs from patients with different diseases including cancer, and the method could make a significant difference in routine testing and early detection, especially considering the relatively rapid readout (<20 min). However, while it is generally well established that EVs carry information about diseases such as lung,^8^ esophageal,^66^ pancreatic,^67^ and breast^68^ cancers, there is no robust consensus on the most reliable markers with multiple candidates reported. It is therefore necessary to comparatively test several candidate markers on biopsies from healthy, precancerous, and malignant cancer patients. If successful, this would fully validate the technology and open the door for testing other potential diseases with this method.

## Conclusion

In this study, we show that the viscoelastic properties of saliva are scale-dependent, with the liquid behaving as a Newtonian fluid at the nanoscale. This is due to the comparatively large scale of the dissolved biopolymers and cell materials that create a slow-evolving mesh through which water and small molecules can move freely. Using microrheology, we demonstrate that tracers smaller than ∼25 nm can diffuse freely provided they do not interact with the biomesh. We exploit this finding to achieve quantitative mechanically based detection of model lipid nanovesicles with a specific biomarker directly inside drops of unprocessed saliva. We illustrate the potential of the technique to detect specific subpopulations of EV based on proteic markers. More work is needed to independently benchmark the technique and confirm the detection of specific EVs. The fact that the detection method is mechanical could also be further exploited to sense pathological variations in the EVs’ mechanical properties,^69^ for example using higher vibration eigenmodes of the lever.^12,27^ Finally, although the focus of the present study is on EV quantification motivated by cancer detection, the method can be in principle applied to the detection of any kind of nano-objects in suitable complex fluids, from viruses to nanoparticles and toxins. It could be particularly useful where samples are limited in quantity or where a relatively rapid answer is needed, for example, the analysis of toxicity changes and pollutant level after each treatment step in wastewater recovery.

## Supporting Information Available

Example thermal spectra of AFM cantilevers in air, water, and saliva; discussion of storage and loss moduli from standard rheological measurements; characterization of the microrheology tracers; details about the acquisition and analysis of the mass uptake data; characterization of the cantilevers’ functionalization; disucssion of the baseline noise and negative controls; Figures S1–S8 and Table S1 (PDF)
