# Supplementary material for: Quantitative Detection of Biological Nanovesicles in Drops of Saliva Using Microcantilevers
Source: ACS Appl Mater Interfaces. 2023 Dec 29;16(1):44–53. doi: 10.1021/acsami.3c12035 (PMC10788824; doi:10.1021/acsami.3c12035)
Supplement: Supplementary file 1 — am3c12035_si_001.pdf [file am3c12035_si_001.pdf]

Supporting Information

Quantitative detection of biological nanovesicles in drops of saliva using microcantilevers

Clodomiro Cafolla[1], James Philpott-Robson[1], Aaron Elbourne[2] and Kislon Voitchovsky[1]*

[1]Department of Physics, Durham University, Durham UK, DH1 3LE, UK

[2] School of Science, STEM College, RMIT, University, Melbourne, VIC 3001, Australia

*Email: kislon.voitchovsky@durham.ac.uk

Content:

1. Representative thermal spectra of AFM cantilevers in air, water and saliva
   (Fig. S1)

2. Storage and loss moduli for water and saliva from standard rheological measurements
   (Fig. S2)

3. Size characterisation of the microrheology tracers
   (Fig. S3 and Table S1)

4. Acquisition and analysis of the mass uptake data
   (Fig. S4 and S5)

5. Choice of cantilever and optimisation of the detection

6. Characterisation of the cantilevers' functionalisation
   6.1. Tuning spectra
        (Fig. S6)
   6.2. Scanning electron microscopy
        (Fig. S7)

7. Baseline noise and negative controls
   (Fig. S8)

8. Supporting references

1. Example Thermal spectra of AFM cantilevers in air, water and saliva

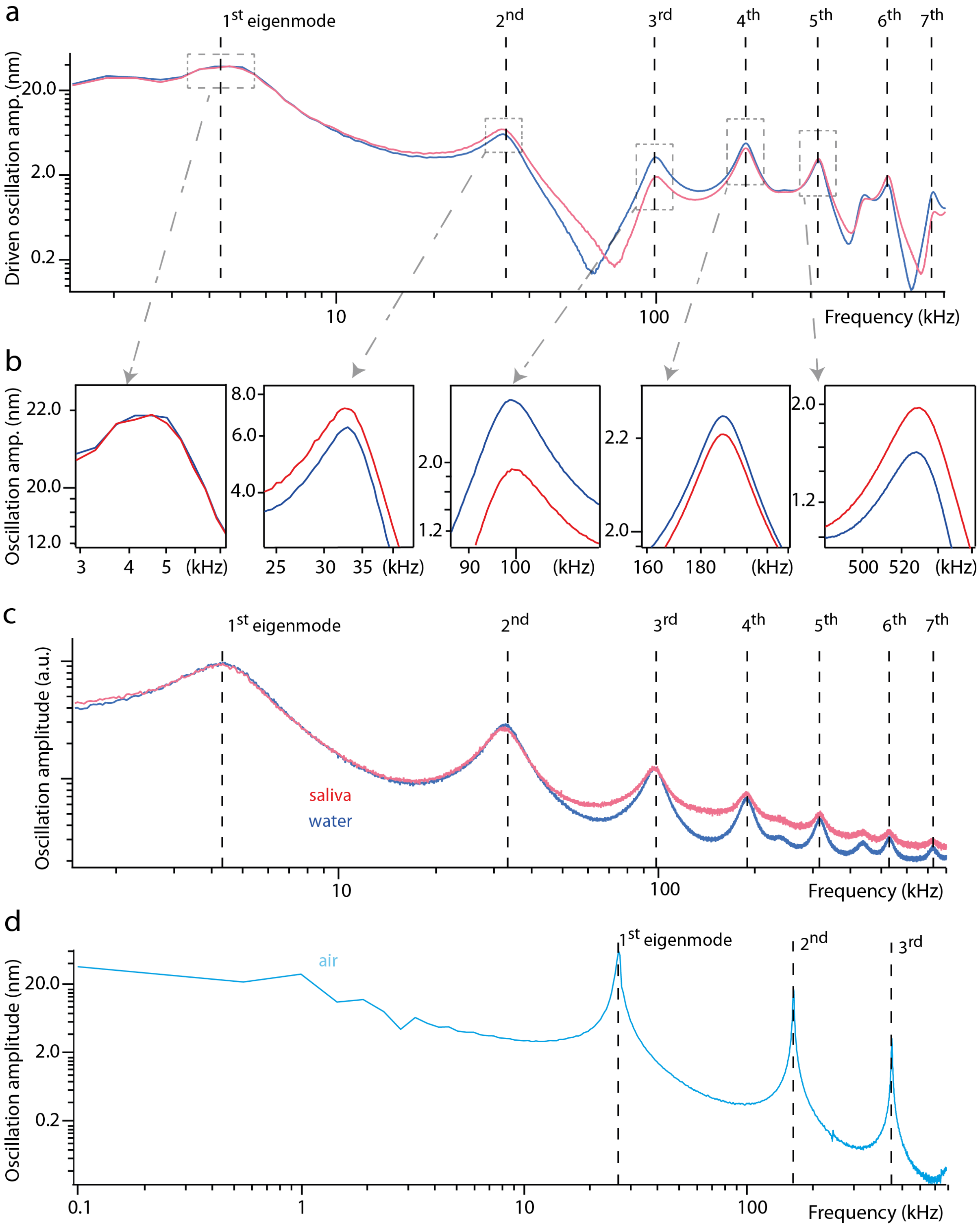

Fig. S1. Dynamic behaviour of a vibrating microcantilever in water and saliva. The externally driven frequency spectrum (tuned) (**a-b**) and the thermal spectrum (**c**) of a 200 µm AFM cantilever (RC800 PSA, long lever) does not perceive any difference between water and saliva within experimental error. This is clear in the magnified and vertically aligned eigenmode resonances (**b**). For comparison, the thermal frequency spectrum of the same cantilever is also shown in air (**d**).

The motion of a cantilever vibrating is not significantly affected by the surrounding liquid medium when vibrating with small oscillation amplitudes. This is the case both when the cantilever is subject to a driving force by photothermal excitation (Fig. S1a-b) and when the cantilever can freely oscillate in the medium of interest (Fig. S1c).
For comparison, Fig. S1d shows the frequency response of the cantilever in air. Within this medium, only 3 eigenmodes can be observed due to the experimental limitations of the AFM lock-in amplifier.
Using the method described in ref.[1], we can reconstruct the saliva viscosity as experienced by the vibrating cantilever. The analysis is based on the use of two consecutive eigenmode resonance frequencies for the cantilever immersed in air, in a reference fluid (here, water) and in the fluid of interest (here, saliva). We apply the method twice to two sets of consecutive eigenmodes: we first apply the method to the 1st and 2nd eigenmodes, and then to the 2nd and 3rd eigenmodes.

2. Storage and loss moduli for water and saliva from standard rheological measurements

Using a standard macroscopic shear rheometer (Advanced Rheometer model AR 2000, TA Instrument, New Castle, DE, USA), the viscoelastic properties of water and saliva were comparatively quantified. This is expressed in the form of the storage and loss moduli of the measured liquid [2,3]. The storage modulus, G', quantifies the elastic response of the sample to an applied shear force. It is defined as the stress/strain ratio measured exactly in phase with applied shear. The equivalence for a vibrating microcantilever would be the force opposing the vibration acting in phase with the oscillation driving the cantilever vibration. The loss modulus, G'', quantifies the viscous response of the sample to an applied shear force. It represents the energy dissipated into the liquid and is defined as the stress/strain ratio measured at a 90° phase lag from the applied shear. Similarly, for a vibrating microcantilever this is equivalent to the force opposing the vibration but with a 90° phase lag from the driving vibration.

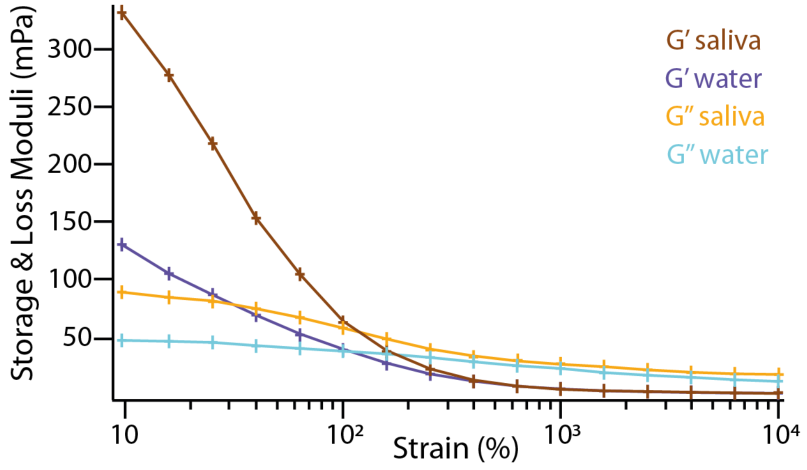

Fig. S2. Storage and loss moduli for water and saliva as a function of the applied strain. The non-Newtonian macroscale behaviour of saliva in comparison to water. Here, the experiments are performed by standard shear rheology. The non-Newtonian response of saliva to the strain is due, in particular, to its elastic component which can be ascribed to the polymers and nanoparticles naturally present in the bodily fluid[4,5]

3. Size characterisation of the microrheology tracers

The silica tracers were measured by dynamics light scattering (DLS) first in pure water and, after coating with DOPC lipids, in PBS. Representative DLS spectra are shown in Fig. S3 for each of the tracer size, with the associated size distribution characteristics summarised in Table S1.

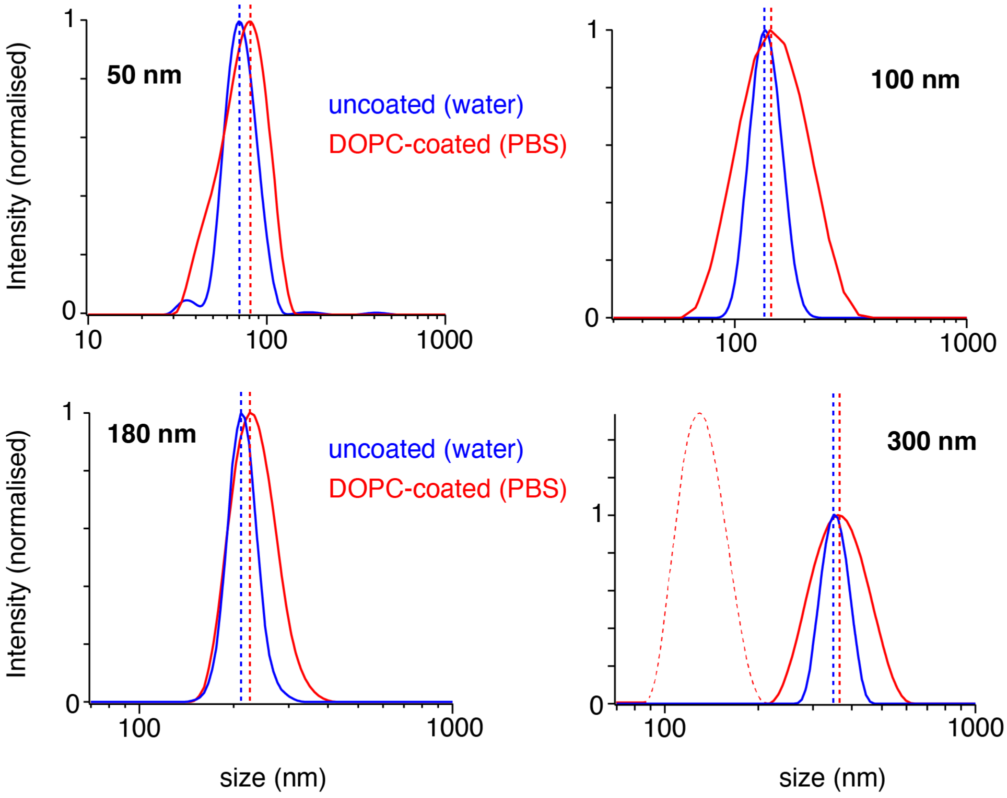

Fig. S3. Representative DLS spectra for the tracers used in the study. The nominal size of the tracer is shown in bold on each set of measurements, with the spectra for uncoated tracers in pure water shown in blue, and those for DOPC-coated tracers in red. In each case, the maximum corresponding to the measured tracer size is normalised to one; this is done to help comparison. The position of each maximum is highlighted with a vertical dashed line, showing slightly broader tracer sizes and distribution width when coated with DOPC (see summary in Table S1). This is expected since the coating may not necessarily be a perfect single bilayer on all tracer particles. In the case of the 300 nm tracers, a concentration of DOPC vesicles higher than that needed for the coating was left in the PBS solution to offer a point of comparison with the tracers: the vesicles are responsible for peak (dashed red) with a maximum at ~150 nm (vesicles extruded through 100 nm pores). This confirms the reliability of the measurements. Note the log scale on the horizontal axis.

Nominal diameter (nm)	DLS diameter (nm)	Error	DOPC coated diameter (nm)	Error
50	73	6	81	6
100	140	10	150	10
180	210	10	220	10
300	360	20	370	10

Table S1. Size of the different tracers (coated and uncoated) obtained by gaussian fitting of the size distributions obtained by DLS. The size difference between the nominal and the DLS results could suggest occasional particle aggregates, but the choice of relying on the latter ensures consistent measurements on each set of tracers, and with the same setup as used for micro-rheology.

4. Acquisition and analysis of the mass uptake data

The mass uptake on the vibrating cantilever is reconstructed from the evolution of its first resonance over time. A frequency sweep is acquired at set time intervals (automated), with the resonance frequency and quality factor (Q-factor) extracted from the resonance amplitude and phase (redundant information). An example of data acquisition is shown in Fig. S4 below.

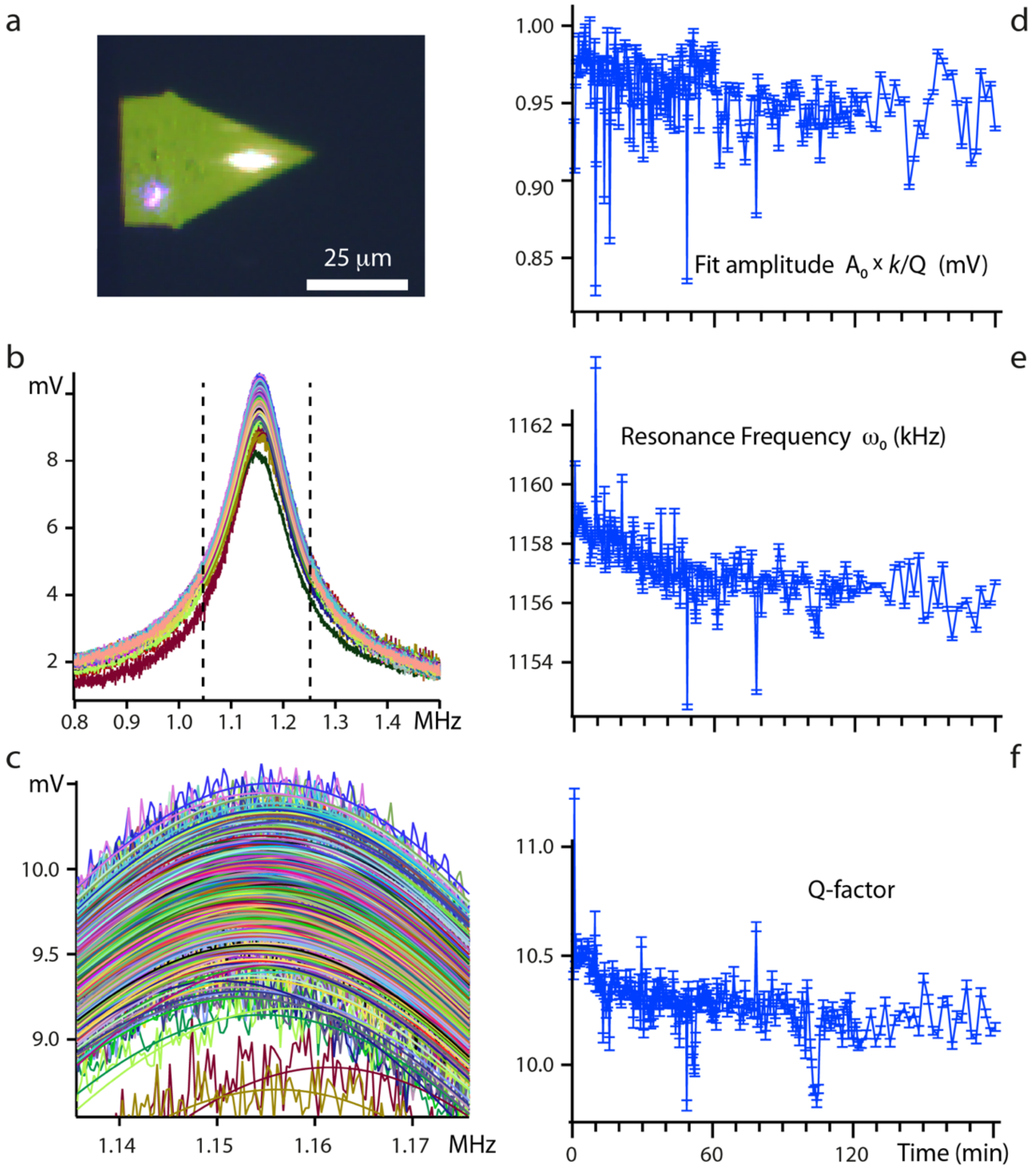

Fig. S4. Representative automated acquisition and analysis of the data for a single experiment. First, the detection laser (**a**) is aligned on the photodiode and acquires a tuned (driven) spectrum (**b**) at set time intervals (typically from every 20 s to every 2 min). Here, an AC55 cantilever (Olympus) is used, driven photothermally (**a**) and the amplitude kept < 0.5 nm at resonance. An interval of frequencies around the resonance is then selected (**b**) and every spectrum simultaneously fitted over the interval with the single harmonic oscillator model (details magnified in (**c**) for amplitude fitting). From the fits, the time evolution of the fit amplitude (related to the real amplitude via k and the Q-factor), the resonance frequency and the Q-factor can be immediately extracted (**d-f**). For all the measurements, the temperature was kept at 25.0 ± 0.1 °C. The same procedure is carried out using the phase of the resonance (see Fig. S5).

There are numerous models to fit the resonance of a cantilever based on its stiffness and geometrical properties[6–11]. The simplest approach, used here, is to use the point mass model (simple harmonic oscillator) and neglect geometrical factors.

The oscillation amplitude of the cantilever is given by:

$$A(\omega) = \frac{A_0}{k\frac{\omega}{\omega_0}\sqrt{\frac{1}{Q^2} + \left(\frac{\omega_0}{\omega} - \frac{\omega}{\omega_0}\right)^2}}, \qquad \text{(S1)}$$

with ω the frequency, Q the quality factor and k the spring constant of the cantilever. The characteristic frequency ω_0 of the oscillator is given by:

$$\omega_0 = \sqrt{\frac{k}{m}}, \qquad \text{(S2)}$$

where m is the mass of the oscillator. By fitting the resonances with Eq. (S1), the time evolution of the different parameters can be extracted.

Since we have a driven, damped oscillator, we cannot use Eq. (S2) to extract the mass of the cantilever but need to correct the resonance frequency to take into account the damping[8]:

$$\omega_r = \omega_0\sqrt{1 - \frac{1}{2Q^2}} = \sqrt{\frac{k}{m}}\sqrt{1 - \frac{1}{2Q^2}}, \qquad \text{(S3)}$$

so that

$$m = \frac{k}{\omega_r^2}\left(1 - \frac{1}{2Q^2}\right), \qquad \text{(S4)}$$

where ω_r is the measured resonance frequency of the cantilever.

Similarly, the shift in the phase, φ, of the oscillation at the resonance can be used to track the relevant parameters even if the amplitude is unreliable using the relation:

$$\tan(\varphi) = \frac{Q(\omega^2 - {\omega_r}^2)}{\omega\omega_r}. \qquad \text{(S5)}$$

Figure S5a shows the phase data φ as a function of ω for each of the spectra shown in Fig. S4b. As shown in Fig. S5b, the phase data are then fitted using Eq. (S5) over the same interval as in Fig. S4c. The fits can be used to extract the evolution of Q and ω_r over time, providing a different route to the same results as those presented in Fig. S4d (apart from the amplitude), and can therefore be used to improve accuracy. From Eq. (S4), the added mass (mass uptake) can be hence quantified over time.

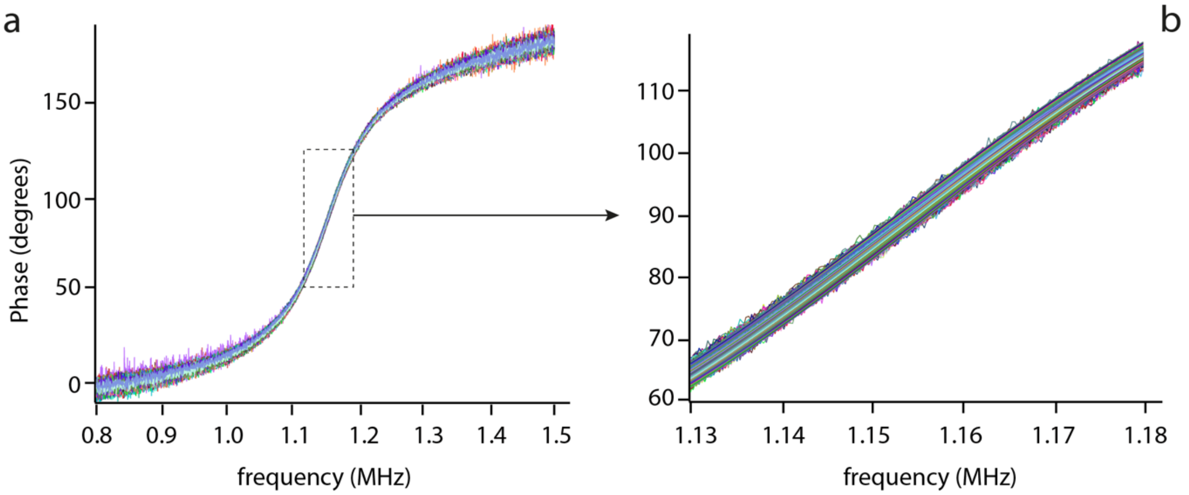

Fig. S5. Analysis of phase information associated with the data presented in Fig S4. The phase variation over the whole width of the resonance as presented in Fig. S4b is shown in (**a**). A subset of the frequency range (dashed rectangle in (**a**)) is used for fitting with Eq. (S5) (**b**). This is done using an automated fitting procedure, yielding the resonance frequency and the Q-factor.

5. Choice of cantilever and optimisation of the detection

The time-dependant methodology and analysis described in the manuscript Methods swas first trialled for a relatively soft and long cantilever (OMCL RC800 PSA, 100 µm x 40 µm x 0.4 µm, with a nominal stiffness of ~0.76 N/m, see Fig. 1), functionalised as described in the main manuscript. The interest of using longer probes with relatively large surface area is the binding of more vesicles in comparison to smaller cantilevers, thereby increasing the added mass. However, softer cantilevers suffer from larger noise levels. From Eqs (S2-S4), the change of mass upon vesicles adsorption can be approximated as[12]:

$$\Delta m \sim k\left(\frac{1}{\omega_{r,0}^2} - \frac{1}{\omega_{r,ves}^2}\right), \qquad \text{(S6)}$$

with $\omega_{r,0}$ and $\omega_{r,ves}$ the resonance frequencies of the cantilever without and with vesicles adsorbed, respectively. Equation (S6) suggests that the best strategy for optimising mass detection consists in using high stiffnesses and large absolute frequency shifts. Working with cantilevers exhibiting high resonance frequencies would also be useful to improve the signal to noise ratio. We therefore resorted to using AC55 cantilever (Olympus, nominal stiffness of approx. 50 N/m and resonance in water/saliva of approx. 1.2 MHz).

6. Characterisation of the cantilevers' functionalisation

6.1. Tuning spectra

As clear from Supporting section 4, the characteristics of cantilevers' tuning spectra contains quantitative information about its mass uptake from adsorbing material from the solution. Figure S6 presents a representative set of spectra acquired for the same cantilever at the different functionalisation stages. As expected, the resonance frequency is significantly higher in air than in liquid due to the lower viscous drag. The spectra acquired for the unfunctionalised cantilever in pure water are similar with a small down-shift of the resonance frequency in PBS (inset in the lower graph of Fig. S6) suggesting the adsorption of buffering ions[13]. The effect is however hardly visible, and the difference is close to the detection limit. Interestingly, functionalisation with the lipids (including 0.5% biotin) induces an increase in the resonance frequency despite the added mass; this increase reflects the reduced water-surface interactions and hence viscous drag on the vibrating cantilever. This is a clear signature of the role played by the zwitterionic lipid headgroups in limiting non-specific interactions between the cantilever and its surrounding liquid. Adding the avidin (here neutravidin) does not change the cantilever-water interactions but adds mass, predictably reducing the resonance frequency.

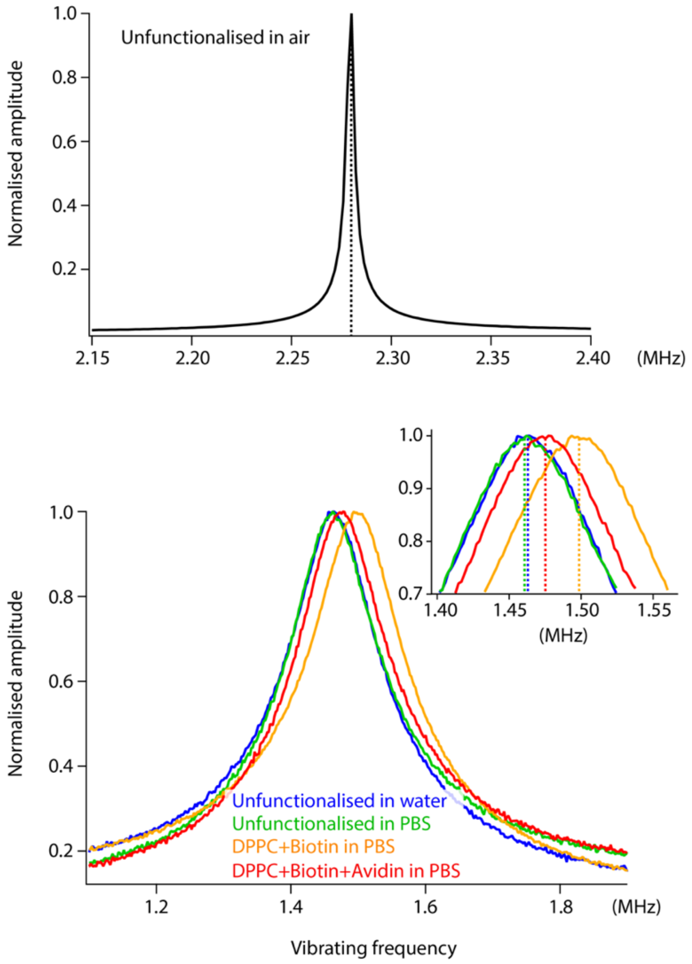

Fig. S6. Tuning spectra of a representative cantilever acquired at each stage of the functionalisation. The unfunctionalised cantilever exhibits a sharp resonance in air at ~2.28 MHz (upper panel). The resonance peak broadens in liquid (lower panel) with a lower resonance frequency than in air, and a barely visible downshift in frequency from pure water to PBS solution (see inset). The functionalisation with the zwitterionic liquid bilayer (+biotin) reduces the cantilever drag in the solution, effectively increasing its resonance frequency. Adding neutravidin however reduces the resonance frequency due to the added mass.

6.2. Scanning electron microscopy

To independently evaluate the impact of the measurements on the cantilever and verify the presence or absence of saliva debris afterwards, we examined selected cantilevers by scanning electron microscopy (SEM) (Fig. S7). Transmission electron microscopy (TEM) can achieve higher resolution including possible visualisation of the functionalisation and adsorbed EVs but this would require cryogenic temperatures to preserve the biological material with sufficient water in glassy state[14] (not available here). Since the success of the functionalisation is established (see previous section), the goal of this section is primarily to inspect possible large aggregates, cells, cell fragments and debris potentially adsorbed to the cantilevers. In comparison to an unfunctionalized cantilever in water (Fig. S7a), no clear difference is visible when the same cantilever is functionalised and then exposed to either PBS (Fig. S7b), saliva with model EVs (Fig. S7c) or saliva for CD9 capture (Fig. S7d). No large debris (> 500 nm) is visible.

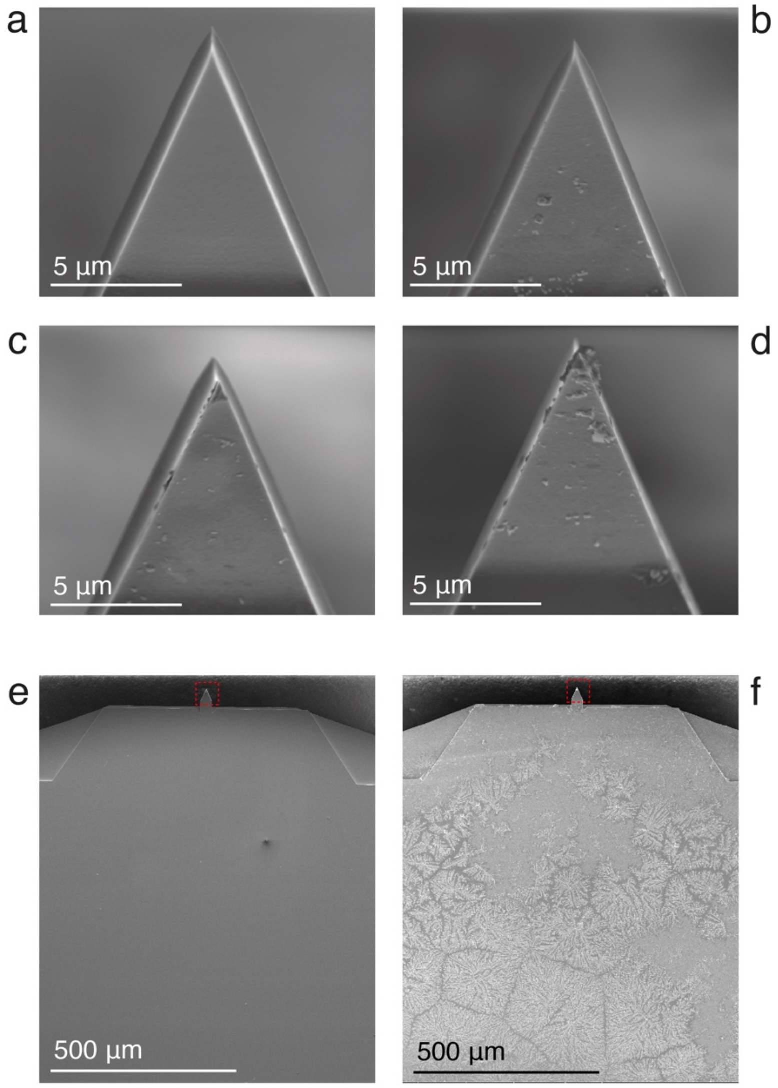

Fig. S7. *Ex-situ* representative SEM images of cantilever used for the experiments. (**a**) Control cantilever cleaned and immersed only in pure water (see the Experimental section in the main text) with no functionalisation. (**b**) Functionalised cantilever (biotin + avidin) immersed only in PBS. (**c**) Functionalised cantilever (biotin + avidin) exposed to saliva containing model EVs for 70 min, and subsequently rinsed with PBS. (**d**) Functionalised cantilever (biotin+avidin+anti-CD9 antibody) exposed to saliva for 70 min, and subsequently rinsed with PBS. The cantilever was used as part of the measurements presented in Fig. 4. (**e**) Chip supporting the cantilever (dashed

red square) used for (**a**), revealing a clean surface. (**f**) Chip supporting the cantilever (dashed red square) used for (**b**), revealing salt crystallisation patters from drying. All the SEM data was acquired in a mild vacuum.

7. Baseline noise and negative controls

For the purpose of the present study, the functionalisation of the cantilevers is performed with commercially available molecules and reagents, and on commercially available cantilevers. The focus is on the proof of concept rather than an optimised functionalisation for routine application.

The functionalisation of the cantilevers with the desired antibody is done though conjugation via a biotin-avidin complex, with both biotinylated lipids and avidin available from multiple commercial providers. The direct exposure of biotin or avidin to raw saliva is however challenging since both can bind enzymes and molecules naturally present in the saliva (see e.g. ref[15] and references within). This poses a challenge to set a suitable control for the mass uptake experiments with a significant variability between baseline measurements in a given experiment. In some cases, the control experiment exhibits relatively low noise and behaves as expected (Fig. 3). In other cases, the controls are particularly noisy although systematically exhibiting a single timescale evolution within error. This is illustrated in Fig. S8a where examples of the noisier control experiments are shown for (i) only the DPPC lipid coating, (ii) the DPPC including the biotinylated lipids and (iii) the same as (ii) but further functionalised with avidin (here, neutravidin). For the sake of the present discussion, 'noise' is defined as the average variation between point measurements taken at consecutive times over a particular set (Fig. S8b).

When using a pure DPPC coating, the control is generally well-behaved with limited noise and mass uptake. It should be noted that the example selected here is among the poorest control observed in this study, with the controls for the measurements in Fig. 4 significantly better. Nevertheless, the expected single slow timescale is clearly visible and below the typical mass uptake for targeted detection. When using exposed biotin, the noise level can increase, but the issue depends on the saliva sample. Fitting the time evolution with a single exponential did not return any meaningful conclusions due to the high level of noise. Finally, the exposed avidin induced the highest noise level ever observed, with a mass uptake comparable to the that of an intended target in saliva for the data shown in Fig. S8. We emphasise that biotin and avidin controls are more prone to noise, but this depends on the saliva sample and is by no mean systematic.

Although this is problematic for developing suitable controls, it is important to note that when fully functionalised with an anti-tetraspanin (CD9 or CD81) antibody, the noise level of the measurements systematically decreases to a level comparable to that of pure DPPC (Fig. S8b). This is consistent with the interpretation of exposed biotin and avidin being problematic as negative controls, rather than a failure of targeted detection. This is likely due to biotin and avidin being able to bind to several biomolecules and enzymes present in the saliva[15]. This argument is supported by the comparable mass uptake between negative DPPC control and immune-functionalised cantilever. This also suggests that the noise level of a given detection trace can be used as a basic quality control for both targeted detection and control measurements. This last point is illustrated in Fig. S8c which represents a failed attempt at a measurement comparable to that in Fig. 3 due to undesired binding of biomaterial on the biotinylated + avidin cantilever. First, the noise level is considerable (> 100 pg), second the fouling material interferes with the operating laser, leading to inconsistent readings between 100-160 mins. Binding of large cell fragments or bio-aggregates tend to show sudden and dramatic variations in mass uptake.

For these reasons, we resorted to using pure DPPC coating as the negative control. This ensures the most consistent and reproducible controls (Fig. 4) but may not be directly comparable to the measurements with the functionalised cantilevers. Better controls will therefore have to be established in the future, something that can be achieved by bypassing the biotin-avidin linkers and relying on direct chemical binding of the intended marker to the cantilever.

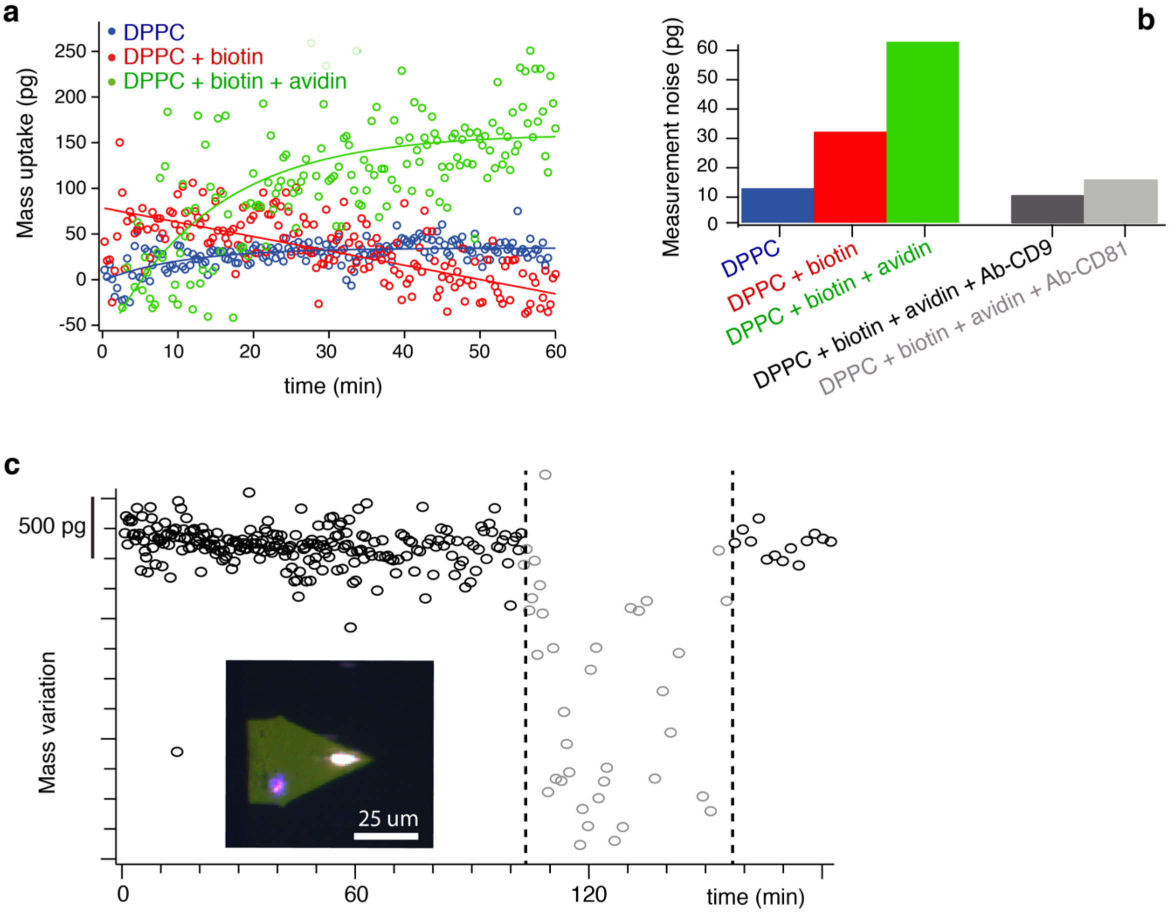

Fig. S8. Comparison of possible cantilever functionalisation for control experiments. (**a**) Three different functionalisation approaches are compared in raw saliva: pure DPPC lipids, DPPC containing biotinylated lipids, and DPPC with biotinylated lipids further functionalised with avidin (here, neutravidin). In each case, the data is fitted with a single exponential curve without imposing any constraint. The data and the fit both confirm a single, slow (> 20 min) timescale, but the significant noise levels make the fitting unrealistic for the biotinylated samples, likely due to fouling. (**b**) The noise level of each measurement can be quantified, here as the average variation between two consecutive points in the temporal mass uptake evolution. For comparison, the noise level of the noisiest data shown in Fig. 4 (before average) is also given. (**c**) The problem of fouling with biotinylated + avidin cantilevers is immediately visible from the increased noise level in the readout (> 100 pg), resulting in the uptake of large biological fragments interfering with the photothermal laser (blue spot in inset) and unreliable reading (between dashed lines) until the laser is readjusted. The measurement shown in (**c**) is performed in saliva containing 0.1 µg/ml of model vesicles solution and using the same control (biotin + avidin) as in Fig. 3. The noise level is particularly high.

8. Supporting references

(1) Payam, A. F.; Trewby, W.; Voïtchovsky, K. Simultaneous Viscosity and Density Measurement of Small Volumes of Liquids Using a Vibrating Microcantilever. *Analyst* **2017**, *142* (9), 1492–1498.
(2) Mezger, T. G. *The Rheology Handbook*, 5th Ed.; European Coatings Library; Vincentz Network: Hanover, 2020.
(3) Doi, M. *Soft Matter Physics*; Oxford University Press, 2013.
(4) Zare, Y.; Yop Rhee, K. Experimental Data and Modeling of Storage and Loss Moduli for a Biosensor Based on Polymer Nanocomposites. *Res. Physics* **2020**, *19*, 103537.
(5) Zare, Y.; Rhee, K. Y. Expression of Normal Stress Difference and Relaxation Modulus for Ternary Nanocomposites Containing Biodegradable Polymers and Carbon Nanotubes by Storage and Loss Modulus Data. *Compos. B: Engineering* **2019**, *158*, 162–168.
(6) Van Eysden, C. A.; Sader, J. E. Frequency Response of Cantilever Beams Immersed in Viscous Fluids with Applications to the Atomic Force Microscope: Arbitrary Mode Order. *J. Appl. Phys.* **2007**, *101* (4), 44908.
(7) Chon, J. W. M.; Mulvaney, P.; Sader, J. E. Experimental Validation of Theoretical Models for the Frequency Response of Atomic Force Microscope Cantilever Beams Immersed in Fluids. *J. Appl. Phys.* **2000**, *87* (8), 3978–3988.
(8) García, R.; Pérez, R. Dynamic Atomic Force Microscopy Methods. *Surf. Sci. Rep.* **2002**, *47* (6–8), 197–301.
(9) Rodríguez, T. R.; García, R. Tip Motion in Amplitude Modulation (Tapping-Mode) Atomic-Force Microscopy: Comparison between Continuous and Point-Mass Models. *Appl. Phys. Lett.* **2002**, *80* (9), 1646–1648.
(10) Sader, J. E.; Chon, J. W. M.; Mulvaney, P. Calibration of Rectangular Atomic Force Microscope Cantilevers. *Rev. Sci. Inst.* **1999**, *70* (3967).
(11) Sader, J. E. Frequency Response of Cantilever Beams Immersed in Viscous Fluids with Applications to the Atomic Force Microscope. *J. Appl. Phys.* **1998**, *84* (1), 64-76.
(12) Singamaneni, S.; Lemieux, M. C.; Lang, H. P.; Gerber, C.; Lam, Y.; Zauscher, S.; Datskos, P. G.; Lavrik, N. V; Jiang, H.; Naik, R. R.; Bunning, T. J.; Tsukruk, V. V. Bimaterial Microcantilevers as a Hybrid Sensing Platform. *Adv. Mater.* **2008**, *20* (4), 653-680.
(13) Trewby, W.; Livesey, D.; Voitchovsky, K. Buffering Agents Modify the Hydration Landscape at Charged Interfaces. *Soft Matter* **2016**, *12* (9), 2642–2651.
(14) Mielanczyk, L.; Matysiak, N.; Michalski, M.; Buldak, R.; Wojnicz, R. Closer to the Native State. Critical Evaluation of Cryo-Techniques for Transmission Electron Microscopy: Preparation of Biological Samples. *Folia Histochem. Cytobiol.* **2014**, *52* (1), 1–17.
(15) Lu, C.-S.; Kashima, K.; Daa, T.; Yokoyama, S.; Yanagisawa, S.; Nakayama, I. Immunohistochemical Study of the Distribution of Endogenous Biotin and Biotin-Binding Enzymes in Ductal Structures of Salivary Gland Tumours. *J. Oral Pathol. Med.* **2000**, *29* (9), 445–451.
